# Chronic Neutrophilic Leukemia: A Comprehensive Review of Clinical Characteristics, Genetic Landscape and Management

**DOI:** 10.3389/fonc.2022.891961

**Published:** 2022-04-14

**Authors:** Thomas P. Thomopoulos, Argiris Symeonidis, Alexandra Kourakli, Sotirios G. Papageorgiou, Vasiliki Pappa

**Affiliations:** ^1^Second Department of Internal Medicine, Attikon Hospital, Research Institute, National and Kapodistrian University of Athens, Athens, Greece; ^2^Department of Internal Medicine, University Hospital of Patras, Rio, Greece

**Keywords:** chronic neutrophilic leukemia, myeloproliferative neoplasm, CSF3R, ruxolitinib, allogeneic HSCT

## Abstract

Chronic neutrophilic leukemia (CNL) represents a rare disease, that has been classified among the BCR/ABL-negative myeloproliferative neoplasms. The disease is characterized by marked leukocytosis with absolute neutrophilia and its clinical presentation may vary from asymptomatic to highly symptomatic with massive splenomegaly and constitutional symptoms. CNL prognosis remains relatively poor, as most patients succumb to disease complications or transform to acute myeloid leukemia. Recent studies have demonstrated that *CSF3R* mutations drive the disease, albeit the presence of other secondary mutations perplex the genetic landscape of the disease. Notably, the presence of *CSF3R* mutations has been adopted as a criterion for diagnosis of CNL. Despite the vigorous research, the management of the disease remains suboptimal. Allogeneic stem cell transplantation represents the only treatment that could lead to cure; however, it is accompanied by high rates of treatment-related mortality. Recently, ruxolitinib has shown significant responses in patients with CNL; however, emergence of resistance might perturbate long-term management of the disease. The aim of this review is to summarize the clinical course and laboratory findings of CNL, highlight its pathogenesis and complex genetic landscape, and provide the context for the appropriate management of patients with CNL.

## Introduction

Chronic neutrophilic leukemia (CNL) is a rare disease, with an annual incidence of about 1 new case per million people, a median age at diagnosis of about 65 years and a slight male preponderance. The disease was initially described in 1920 by Tuohy who reported the case of a 58-year-old female patient with persistent polymorphonuclear hyperleukocytosis and massive splenomegaly, who subsequently succumbed to the disease (1). Due to the rarity of the disease and the absence of clear diagnostic criteria, some early described cases are ill-defined; however, an additional well-documented case from France was published in 1932 (2), followed by a paucity of publications for almost 25 years, until the report of a case describing the disease as a myeloproliferative neoplasm (MPN) (3). After the identification of Philadelphia chromosome as a hallmark of chronic myelogenous leukemia (CML), Tanzer et al. were the first to report a patient without Philadelphia chromosome or increased marrow fibrosis but high Leucocyte- or Neutrophil- Alkaline Phosphatase (LAP or NAP) score. Notably, they used the term “Chronic Neutrophilic Leukemia” to describe the disease (4). Additional case reports or small series of patients were published thereafter that have delineated this syndrome, although in some of them the authors have described the disease as a variant of CML (5–12).

CNL is a clonal hematopoietic stem cell disorder, and this has been documented early, as soon as clonality assays with X-linked restriction fragment length polymorphisms (RFLP) and/or X-inactivation patterns were applied (13, 14). The clonal nature of the disease is also evident by the detection of cytogenetic abnormalities (15–17), although in most cases, patients with CNL have a normal karyotype. From the epidemiological aspect of view the precise etiology of the disease remains unknown and there is only one report, associating CNL with a previous exposure to thorotrast, in a patient who, eventually evolved to acute myeloid leukemia (AML) (18). Moreover, when applying strict molecular criteria for disease characterization, it appears that the median age at diagnosis might be younger (19). Recently, mutations of the *CSF3R* gene have been shown to constitute the driver genetic event for the manifestation of this disease (20), which according to the latest version of the WHO classification of Hematopoietic Neoplasms, is classified as a chronic myeloproliferative neoplasm (21) However, there has been a vigorous debate whether CNL should be classified instead among the myelodysplastic syndromes (MDS) or the overlap myelodysplastic/myeloproliferative (MDS/MPN) neoplasms, similarly to chronic myelomonocytic leukemia (CMML), as it might share several clinical, hematological and cytogenetic features, reminiscent of MDS or MDS/MPN. Recently, criteria for the precise diagnosis of this disease have been proposed by the latest revision of the WHO classification, although some variation in clinical parameters might exist.

## Clinical Manifestations

CNL has substantial clinical and prognostic heterogeneity, which might correspond to the different driver mutations of the *CSF3R* gene. Most patients may go through an asymptomatic or minimally symptomatic period, characterized only by neutrophilic leukocytosis, with or without mild splenomegaly, lasting for several months or even years, before evolving to symptomatic disease. In many cases the disease may be discovered incidentally as unexplained persistent neutrophilia, after excluding any other potential causes as infections or inflammatory and neoplastic disorders, which occasionally can induce extreme hyperleukocytosis (22, 23). Less commonly, patients may complain for fatigue and restlessness, weight loss, night sweats, pruritus, and mild bone pain or may exhibit various hemorrhagic manifestations and recurrent episodes of gout (24). Fever is usually absent or minimal but persistent low-grade fever may be a prominent feature of advanced disease.

Bleeding diathesis, occasionally unrelated or disproportional to the degree of thrombocytopenia or splenomegaly/hypersplenism, is also a prominent feature of CNL, potentially reflecting platelet qualitative abnormalities (5, 7, 25, 26). Severe hemorrhagic manifestations, including recurrent episodes of epistaxis, blood oozing from the site of bone marrow aspiration, gastrointestinal tract hemorrhages and even intracranial hemorrhages have been reported throughout the disease course and may represent a common cause of death in these patients (5, 25, 26). One study of three patients with CNL, demonstrated prolonged bleeding time and abnormal platelet aggregation response to collagen, epinephrine and ADP, whereas in one patient, platelet adhesiveness and adenine content were found decreased (27). Moreover, abnormalities of the vascular compartment might contribute to the hemorrhagic manifestations. There have been several reports of cutaneous leucocytoclastic vasculitis or acute febrile neutrophilic dermatosis (Sweet’s syndrome), occurring in patients with CNL. Therefore, various types of erythematous and/or hemorrhagic skin lesions, occasionally affecting palms and soles, represent a common manifestation of CNL. In some of these cases it is unclear whether the described skin lesions represent an allergic reaction, vasculitis or frank skin infiltration by the leukemic neutrophils, but whatever might be the case, severity of skin lesions runs in parallel with disease activity and severity (28–32).

Despite leukocytosis/neutrophilia patients with CNL are prone to develop severe, life-threatening or even fatal infections from both, common and opportunistic pathogens, such as mycobacteria and various fungi. This could be explained by the defective function of neutrophils as described in the following section. Patients may present with a severe infection, sometimes inducing septic shock (33, 34) or infections may occur frequently, during the course of the disease, and ultimately, they may represent one of the main causes of death (35, 36). It is therefore, noteworthy that in such cases, suspicion for the diagnosis of CNL may be obscured by the concurrent presence of a severe infection and neutrophilic leukocytosis often marked, which might be attributed to the infection.

Hepato-splenomegaly is commonly present. Splenomegaly is found in about 40% of the patients at the time of initial diagnosis and in the majority of them is mild to moderate; however, as disease slightly and steadily progresses, splenomegaly may become severe in almost all patients (10–12). Several cases of massive splenomegaly have been described, rarely requiring palliative splenectomy. When disease is rapidly evolving and white blood cells (WBC) are fast increasing, splenomegaly might become painful. Hepatomegaly is less common and less prominent, whereas lymphadenopathy has occasionally been reported but it is unusual. In some cases, lymph node biopsy has clearly revealed neutrophilic infiltration (37), whereas in some autopsy studies, infiltration by immature neutrophils and by other hematopoietic cells were identified in retroperitoneal lymph nodes, spleen, liver and the kidneys, a finding supporting the proliferative nature of the disease (37, 38). Although, vein thrombotic episodes in association with CNL have been rarely reported (39, 40), a recent description of the clinical course of the disease in 19 patients, reported a history of thrombosis in about half of the patients (41).

## Laboratory Findings

The peripheral blood smear is characterized by persistent mature neutrophilic leukocytosis, with about 80-95% of the enumerating cells being neutrophils or bands. Left shift is absent or minimal and only occasional myelocytes-metamyelocytes are observed. Notably, CNL is characterized by the absence of monocytosis, eosinophilia or basophilia. In some cases, leukocytosis/neutrophilia might be severe, exceeding 100 x 10^9^/L and reaching up to 500 x 10^9^/L. Neutrophils appear morphologically normal by both, light and electron microscopy and in the majority of cases contain abundant primary and secondary granules (8). Presence of Döhle bodies and neutrophil inclusions have been reported rarely; however, dysgranulopoiesis is not consistent with the diagnosis of CNL (21). Notably, reported cases of CNL presenting with dysplastic morphological features, particularly in more advanced or long-standing cases, including abnormal chromatin clumping, poor granulation, cytoplasmic vacuolation, microtubular inclusions (42), ringed-shape nuclei (42, 43), are now considered to represent cases of atypical CML rather than genuine CNL cases

Neutrophils and granulocytes of patients with CNL usually have an activated phenotype, but they might also exhibit several functional abnormalities and are less viable in stress conditions. The enzymatic equipment of neutrophils is normal, as this can be revealed by the appropriate cytochemical stains. Myeloperoxidase is strongly positive as is also LAP, whose elevated score has been used to distinguish this disease from BCR/ABL positive CML, in which LAP is very low to completely absent (6, 7, 11, 12). Some studies have reported reduced lysozyme and β-glycuronidase content in the neutrophils (8, 37) but these enzymes are highly released from clonal neutrophils, following stimulation. Neutrophil motility, deformability and chemotaxis have been found abnormal and the respiratory burst is usually impaired, with reduced superoxide anion production in response to various stimuli, as well as decreased cytosolic C kinase activity (44, 45). Other studies have demonstrated normal phagocytosis and bactericidal activity and normal nitroblue tetrazolium reduction assays suggesting that these neutrophils are found in an abnormally activated status (46). Bactericidal activity, however, has also been reported to be decreased and the same patients exhibited decreased granulocytic clonogenic activity from both, peripheral blood and bone marrow, compared to normal subjects (47). In one study, several functional neutrophils tests were found normal, however neutrophils of CNL patients produced significantly lower amounts of leukotriene B4 (48). In another multifunctional study the authors found reduced serum G-CSF and GM-CSF levels and inadequate *in vitro* neutrophil stimulation, induced by these cytokines. They also demonstrated impaired STAT3 and MAP kinase downstream signal, despite the intact expression of both G-CSF- and GM-CSF receptor and they suggest a potential dysregulation of the intracellular part of the receptor(s), although they were unable to show any mutation of this domain (49).

Mild to moderate anemia might be present, in most cases normochromic-normocytic sharing the features of the anemia of chronic diseases (12), whereas cases with macrocytic anemia have also been reported, despite the very high serum B12 levels, reflecting underlying dyserythropoiesis, as shown by the report of impaired erythroblastic colony formation in patients with CNL (7).

Platelet count may be normal, reduced or less commonly, increased. Morphologically, platelets may appear normal, but giant platelets and platelets with poor granulation, reminiscent of storage pool disorders have been observed (11, 12). Deteriorating thrombocytopenia might be a feature of disease transformation towards AML. Rarely, nucleated red blood cells can also be identified. In a recent short report, numerous ringed sideroblasts were found in the marrow of a patient with CNL (50).

Bone marrow findings are more reminiscent of a myeloproliferative neoplasm, with almost 100% cellularity and fat disappearance, extreme granulocytic hyperplasia without maturation arrest and absence of monocytosis, monocyte precursors and basophilia (11, 12). The myeloid/erythroid ratio is usually higher than 10, but blast cells are not increased, unless the disease has entered an accelerated phase and evolves towards AML (51). Megakaryocytic hyperplasia is also common, and in most cases megakaryocytes appear morphologically normal and mainly mature and hyper-lobulated, i.e. with higher ploidy as compared to MDS or typical BCR/ABL-positive CML (27); however, megakaryocyte dysplasia is absent or minimal. Moreover, minimal fibrosis might be present in the bone marrow of patients with CNL but should not exceed a grade of 1+ (21). As a result of increased cellular turnover, pseudo-Gaucher cells may be found in the marrow (52).

The main findings of patients’ biochemical profile consist of elevated serum LDH and uric acid levels, reflecting the increased hematopoietic cell turnover. Serum B12 and transcobalamin-I and -III, that are released in circulation by the mature cells of granulopoietic lineage, are usually elevated. Serum alkaline phosphatase and γ-glutamyl-transpeptidase might also be found increased, reflecting liver infiltration by hematopoietic cells. Markers of acute phase reaction are usually normal at the time of initial diagnosis but may become abnormal in advanced stages, associated with marked leukocytosis or in cases of disease progression. Serum G-CSF levels are not routinely estimated, but if performed, they are found suppressed. In milder cases of CNL differential diagnosis should exclude a leukemoid reaction attributed to various underlying diseases and conditions, and other types of myeloproliferative disorders. A practical diagnostic approach together with the diagnostic criteria of CNL are shown in **Table 1**.

**Table 1:** Diagnostic work up and diagnostic criteria for CNL.

Work up	Diagnostic criteria
Confirm persistent leukocytosis/neutrophilia	Repeated blood counts should confirm WBC >25 x 10^9^/L in repeated evaluations
Exclude all reactive or secondary causes of neutrophilia	Complete survey must not reveal any cause of secondary neutrophilia and if revealed, a *CSF3R* mutation should also be present
Evaluate peripheral blood findings and morphology	>80% of the WBC should be neutrophils or bands, with less mature forms <10% and blasts <1%
Evaluate the clinical and biochemical profile	Hepatosplenomegaly is common, and serum LDH, uric acid, B12, and liver cholestatic enzymes are usually increased
Evaluate cytochemical profile	LAP score is elevated or markedly elevated
Evaluate bone marrow findings and morphology	Marrow cellularity is highly increased with granulocytic hyperplasia without evident dysplasia or excess of blasts
Evaluate bone marrow histology and immunophenotype	A clear myeloproliferative syndrome pattern without an increase of monocytes, eosinophils, basophils or mast cells
Exclude BCR/ABL positive Chronic Myelogenous Leukemia	PCR for BCR/ABL transcripts should be negative and karyotype should not exhibit Ph chromosome
Exclude BCR/ABL negative myeloproliferative neoplasms	Diagnostic criteria for polycythemia vera, primary myelofibrosis and essential thrombocythemia should not be confirmed
Exclude chronic myelomonocytic leukemia	The absolute monocyte count in the blood should be <1 x 10^9^/L
Exclude a classical myelodysplastic syndrome	Dysplastic changes should be absent
Exclude atypical BCR/ABL-negative chronic myelogenous leukemia	Dysplastic changes should be absent. Multilineage dysplastic changes and >10% immature cells in the PB are prominent in aCML. *CSF3R* mutation should be demonstrated
Perform direct molecular characterization of the disease	There should be a mutation in the *CSF3R* gene, but with NGS additional mutations may also be revealed
Investigate for presence of commonly coexisted conditions	Extramedullary infiltration, vasculitic syndromes or plasma cell dyscrasias might be present. *CSF3R* mutations necessary to be confirmed

## Differential Diagnosis

In order to establish the diagnosis of CNL, other cases of reactive leukocytosis should be excluded. Occasionally CNL has been reported to coexist with lymphoid neoplasms (53), but the most striking association has been with various plasma cell dyscrasias, such as monoclonal gammopathy of undetermined significance (MGUS) (39, 54–58), and mainly multiple myeloma. There have been several cases of patients reported to have concurrently or consecutively, these two, apparently different hematological dyscrasias (28, 59–71). However, most of these cases represent a neutrophilic leukemoid reaction, potentially mediated by cytokines produced by the clonal plasma cells. Notably, these cases cannot fulfill the diagnostic criteria for CNL (72). Nonetheless, in the presence of a plasma cell dyscrasia, clonality must be demonstrated by cytogenetic or molecular in order to establish the diagnosis of CNL the diagnosis of CNL (21).

As per the 2016 WHO classification, other MPN and MDS/MPN should also be excluded. Classical MPN, namely polycythemia vera, essential thrombocytosis, and primary myelofibrosis can be easily excluded by the absence of the characteristic morphological and molecular features of the latter. Similarly, the absence of *BCR/ABL* fusion gene invariably precludes the diagnosis of CML. Regarding chronic myelomonocytic leukemia, presence of persistent absolute monocytosis and dysplastic features distinguish this entity from CNL (21). Undoubtedly, the most challenging aspect in differential diagnosis is to distinguish CNL from atypical chronic myeloid leukemia (aCML). Presence of prominent dysplasia in >10% of cells, as well as, a more prominent immature granulocytosis favor the diagnosis of aCML. Notably, presence of *CSF3R* mutations cannot be used for differential diagnosis between aCML and CNL, as there have been several cases of aCML harboring *CSF3R* mutations (73).

## Pathogenesis

### Gene Mutations

#### CSF3R

The granulocyte colony stimulating factor (G-CSF), a cytokine primarily produced by endothelial cells, fibroblasts and macrophages, is the major regulator of both basal and emergency granulopoiesis. G-CSF is crucial for commitment of myeloid cells towards granulocytic differentiation; concomitantly, G-CSF accelerates maturation of metamyelocytes into mature neutrophils, prolongs the survival of neutrophils and their progenitors, and enhances neutrophil function (74).

**Figure 1:**
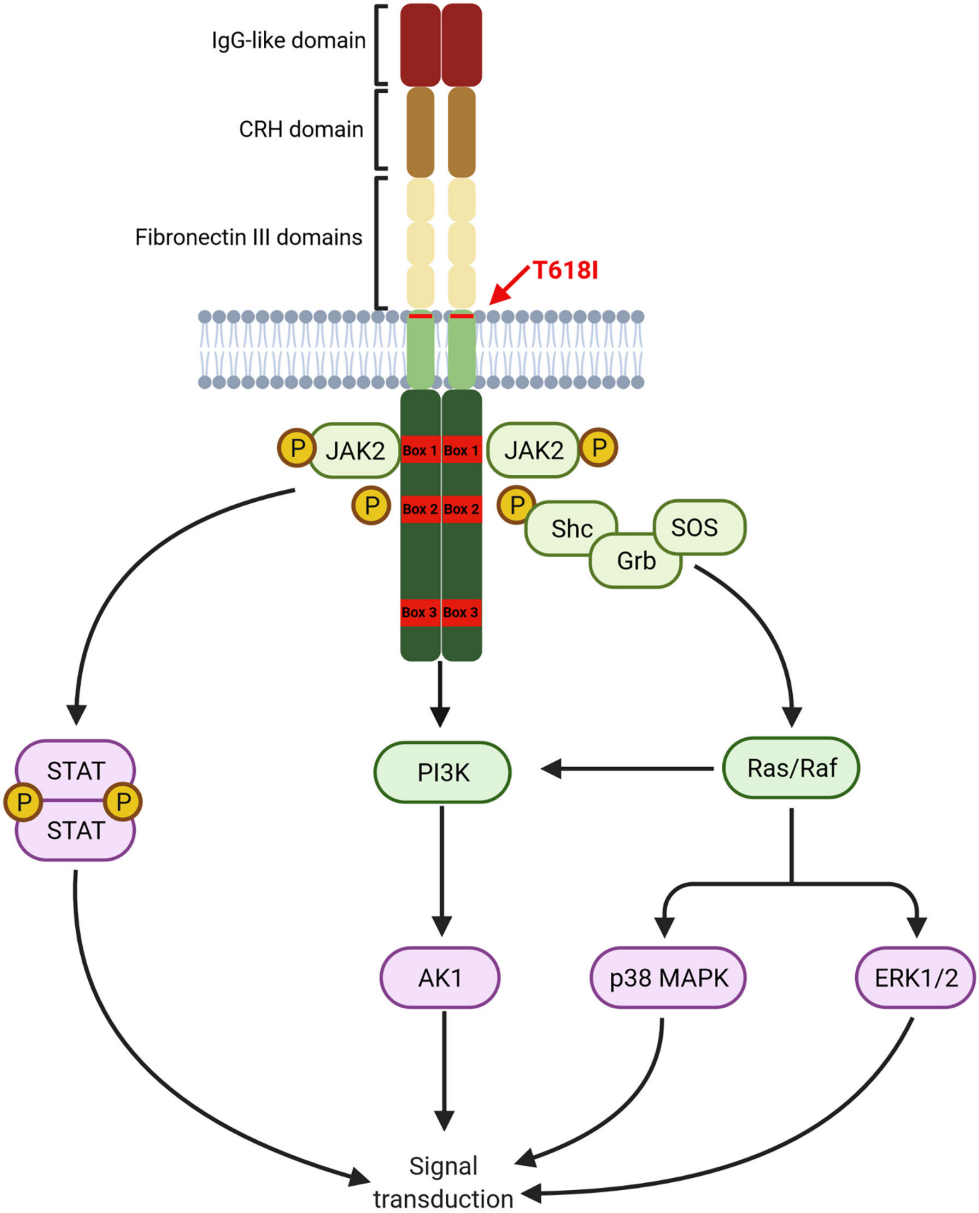
Structure of CSF3R. Proximal transmembrane T618I mutation induced a G-CSF independent activation of receptor homodimers leading to constant signal transduction primarily through the JAK/STAT pathway. Other pathways, namely PI3K/AKT and ERK1/2 are also constantly activated (*Created with BioRender.com*).

G-CSF exerts its actions by binding to the receptor CSF3R on myeloid cells, which consists of a single polypeptide chain with great homology to other cytokine receptors. CSF3R extracellular domain, comprising of an immunoglobulin-like (IgG) domain followed by a cytokine receptor homology domain (CRH) and three fibronectin III (FNIII) domains, plays a crucial role in receptor activation. The intracellular domain contains two proximal motifs termed Box 1 and Box 2 that are critical for signal transduction as they bind to JAK, whereas the distal C-terminal domain drives differentiation and transduction of phagocytic signals in mature neutrophils. C-terminal domain also contains Box 3, which acts as a negative regulator of G-CSF signaling (75). A graphical representation of the CSF3R structure is provided in **Figure 1**.

Binding of G-CSF to CSF3R induces homodimerization of the receptor leading to the activation of downstream pathways. The proximal domain activates mostly the JAK/STAT pathway leading to proliferation and differentiation. The PI3K/AKT pathway is also activated by a LYN kinase, also interacting with the proximal domain, leading to increased survival. Other pathways, particularly the MAPK/ERK are activated by interaction with more distal residues in the intracellular domain.

Given the importance of G-CSF in granulopoiesis, *CSF3R* mutations have been associated with the pathogenesis of various diseases. Extracellular domain missense mutations conferring refractoriness to G-CSF have been identified in severe congenital neutropenia (SCN) and chronic idiopathic neutropenia (CIN) (76). Nonsense or frameshift mutations in the extracellular domain, also found in SCN patients, promote ligand-independent binding to the full-length CSF3R that appears to suppress G-CSF-mediated signaling (77). On the other hand, intracellular domain mutations, mostly of nonsense nature, result in a truncated receptor with normal affinity for G-CSF, but a higher proliferation and lower differentiation potential in response to G-CSF. These mutations are almost exclusively found in patients with SCN who develop MDS or AML.

Proximal transmembrane mutations, namely T618I and T640N, promote the ligand-independent activation of pre-existing dimers of CSF3R and lead to low level, sustained activation of all downstream pathways, that cannot be terminated by internalization of the activated CSF3R, that usually follows the activation of wild-type CSF3R (78). These mutations are found to be particularly enriched in patients with CNL.

In their pivotal trial in 2013, Maxson et al. demonstrated that 8 of 9 CNL patients (89%) harbor *CSF3R* mutations. Notably, all of them had proximal transmembrane mutation, most commonly T618I, whereas an additional truncating intracellular mutation was found in three patients as a compound mutation (20, 79). The results of Maxson et al. have been subsequently validated by Pardanani et al. who demonstrated a *CSF3R* T618I mutation in 83% of patients with WHO-defined CNL. Most strikingly, this mutation was virtually absent from cases of atypical CML or other myeloproliferative neoplasms, highlighting that *CSF3R* T618I could serve as a highly sensitive and specific marker for CNL (80). The oncogenic potential of this mutation has been demonstrated in a murine model, as transplantation of hematopoietic cells harboring the T618I mutation sufficed for the development of a lethal myeloproliferative neoplasm resembling CNL (81). On the other hand, truncating mutations seem not to be oncogenic; however, they might act synergistically with proximal mutations to drive leukemogenesis, potentially through enhanced MAPK signaling, as shown by *in vitro* studies by Rohrabaugh et al. Notably, although compound mutations might confer resistance to JAK or SRC kinase inhibition by ruxolitinib and dasatinib respectively, cell lines harboring compound mutations remain sensitive to MEK inhibitors, such as trametinib.

#### Other Pathogenic Mutations

**Figure 2:**
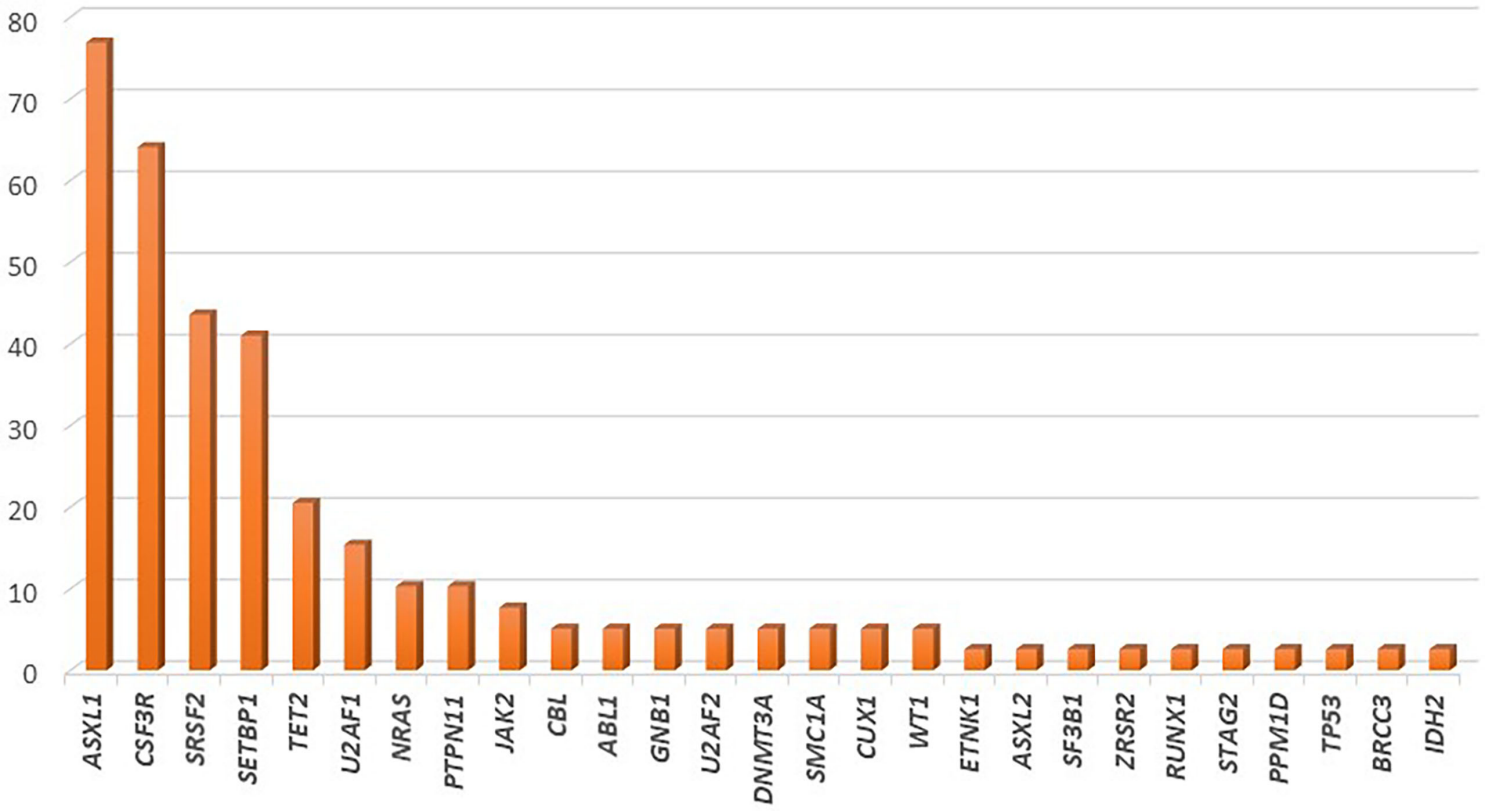
Frequency of gene mutations in chronic neutrophilic leukemia, as demonstrated by application of next generation sequencing in a cohort of 39 patients.

Several recent studies have tried to elucidate the complex genetic landscape of CNL. In most cases, additional mutations in genes commonly affected in myeloid malignancies have been observed; however, their frequency has been highly variable among cohorts. The most commonly affected genes are those involved in epigenetic and transcriptional regulation (*ASXL1*, *TET2*), in the assembly of the spliceosome (*SRSF2*, *U2AF1*), as well as mutations in genes such as *SETBP1*. Notably, mutations in genes involved in the cell-signaling pathways, such as *JAK2* and *NRAS* are scarcely found (82). The frequency of gene mutations in CNL is graphically represented in **Figure 2**.

In the largest cohort of 39 CNL cases, mutations of *ASXL1* were found in 77% of cases. This gene, which is involved in histone modification, was notably the most commonly affected gene in CNL, with frameshift or nonsense mutations. Further, *ASXL1* mutations demonstrated variable variant allelic frequencies (VAF), indicating that this genetic event might occur early in the founder clone, or acquired in later subclones in other cases. Phenotypically, patients with *ASXL1* mutations tended to be of older age and presented with higher WBC count, as well as increased needs for platelet transfusions; a trend for worse overall survival was also noted (83). In line with this finding, Elliot et al. demonstrated that *ASXL1* mutations, present in 57% of cases, along with thrombocytopenia, were independent prognostic factors for adverse overall survival (84). *TET2* mutations, have been observed less frequently in CNL (~21% of cases). In most cases these lesions might be acquired at later stages of clonal evolution. Regarding the disease phenotype, the presence of *TET2* mutations correlated with low platelet count, low neutrophil percentage, high monocyte percentage, and bone marrow dysplasia (83). Of special interest is the fact that no cases with concomitant mutations of *TET2* and *SRSF2* have been observed in CNL, as the *TET2*^mut^/*SRSF2*^mut^ genotype is highly specific of CMML (85). Furthermore, *EZH2* mutations have been found relatively frequently in CNL cases, even though their significance remains unclear.

Mutations affecting the spliceosome have also been reported with variable frequency in patients with CNL. *SRSF2* and *U2AF1*, mutated in up to 44% and 15% of patients respectively, are the most commonly affected genes within this group. Similarly to *ASXL1*, these mutations might be acquired in variable timepoints along the clonal evolution of CNL (83). The contribution of these mutations in disease phenotype and prognosis has yet to be assessed.

*SETBP1* has been found to be mutated in approximately 41% of CNL cases. Notably, a high co-occurrence of *SETBP1* mutations with *CSF3R* T618I has been consistently reported, reaching 67% in more recent studies (86, 87). A trend towards inferior overall survival for patients with CNL harboring *SETBP1* mutations has been reported; however, a meta-analysis of the three available studies that assessed the prognostic significance of this gene in CNL demonstrated no association with overall survival (88). Interestingly, *SETBP1* mutations have been associated with a more myeloproliferative phenotype with increased hemoglobin and platelet counts.

### Models of Clonal Evolution

**Figure 3:**
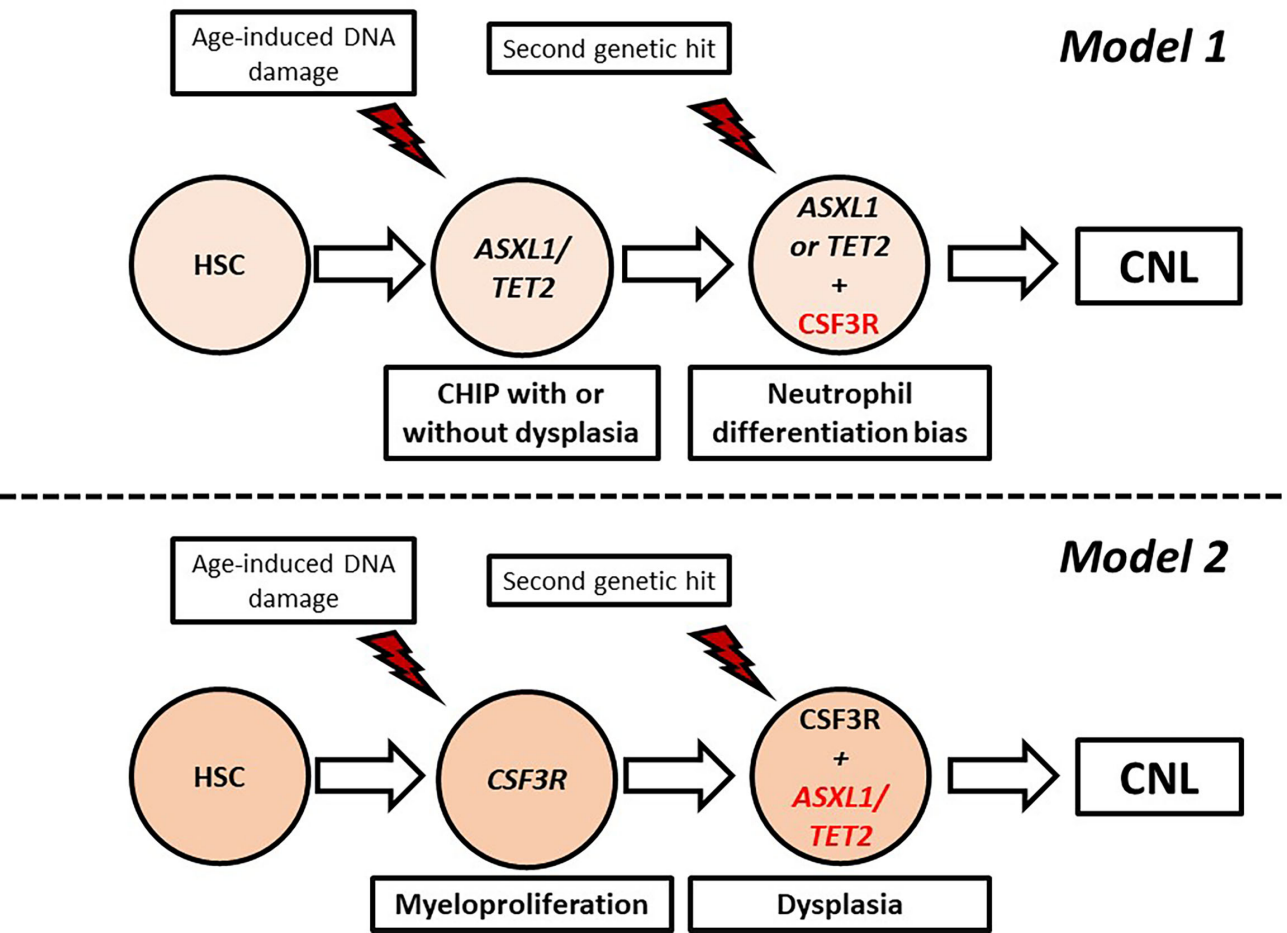
Proposed models of clonal evolution in chronic neutrophilic leukemia. CHIP, Clonal hematopoiesis of indeterminate potential; HSC, hematopoietic stem cell.

CNL demonstrates high genetic heterogeneity with frequent co-occurrence of mutations in different genes with variable VAF. Indeed, in the study of Zhang et al. the median number of mutations per patient was 3.6, indicating that more than three pathways were simultaneously affected in most patients (83). In an attempt to explain the genetic variability of CNL, Maxson et al. suggested that CNL pathogenesis might be a multistep process involving sequential genetic events giving rise to different subclones. At least two models of clonal evolution have been suggested (82). In the first model, a healthy hematopoietic stem cell acquires a mutation associated with epigenetic modification (*ASXL1, TET2*) or spliceosome assembly (*SRSF2, U2A1*), giving rise to a clonal hematopoiesis of indeterminate potential (CHIP) which remains asymptomatic or presents with minimal myelodysplasia. Acquisition of a signaling mutation, most commonly *CSF3R* T618I, offers a neutrophil differentiation bias leading to overt CNL. In the other model, the *CSF3R* T618I arises as a founder mutation in healthy stem cells, causing a highly myeloproliferative phenotype. Owing to the high replication potential, the cells rapidly acquire additional mutations in epigenetic/splicing genes, adding dysplastic features (82). These models are summarized in **Figure 3**.

### Cytogenetic Abnormalities

Most patients with CNL present with normal karyotype at diagnosis. In a series of 40 patients, cytogenetic abnormalities were reported in 32.5% patients. These abnormalities were detected at diagnosis in 20% of patients, or emerged as clonal evolution in the remaining patients. Given their scarcity, no definite conclusions can be drawn regarding the occurrence of cytogenetic abnormalities in CNL; however, deletion of 20q, 11q, 12p, and trisomy 21 might represent nonrandom abnormalities, as they present with increased frequency in other myeloid malignancies (89).

## Prognosis

Overall survival of patients with CNL has been shown to be rather short. In a population-based study combining data from the Surveillance, Epidemiology, and End Results (SEER) program and the National Cancer Database (NCDB), an OS of 1.8 years in SEER and 2.2 years in NCDB was found (90). Similarly, in a series of 40 WHO-defined CNL, median overall survival was 23.5 months; causes of death included intracranial hemorrhage, progressive disease, infections, and leukemic transformation (89). Transformation to AML might occur in 16% of patients at a median of 21 months. Interestingly, several cases of transformation to CMML have been reported (41).

As CNL represents a rare disease, data pertaining to prognostic factors are relatively scarce. Cui et al. demonstrated that WBC count >50 x 10^9^/L represent a negative prognostic factor for OS (86). As mentioned above, Elliot et al. showed that *ASXL1* mutation and thrombocytopenia were independent prognostic factors of inferior OS (84). Most recently, presence of *NRAS*, *ASXL1*, *GATA2*, and *DNMT3A* mutations correlated with a trend of shorter OS, whereas *CBL* mutations predicted a more favorable OS (83). A prognostic scoring system has been suggested by Szuber et al. from Mayo Clinic, incorporating three variables, namely platelet count <160 × 10^9^/L, leukocyte count >60 × 10^9^/L, and presence of *ASXL1* mutation. Thrombocytopenia has been assigned with two points, whereas the other variables with one point each. A two-tier stratification of patients into low-risk (0–1 points) and high-risk (2–4 points), yielded a statistically significant difference in OS (not reached for low-risk vs. 22.4 months for high-risk) (41).

## Management

**Figure 4:**
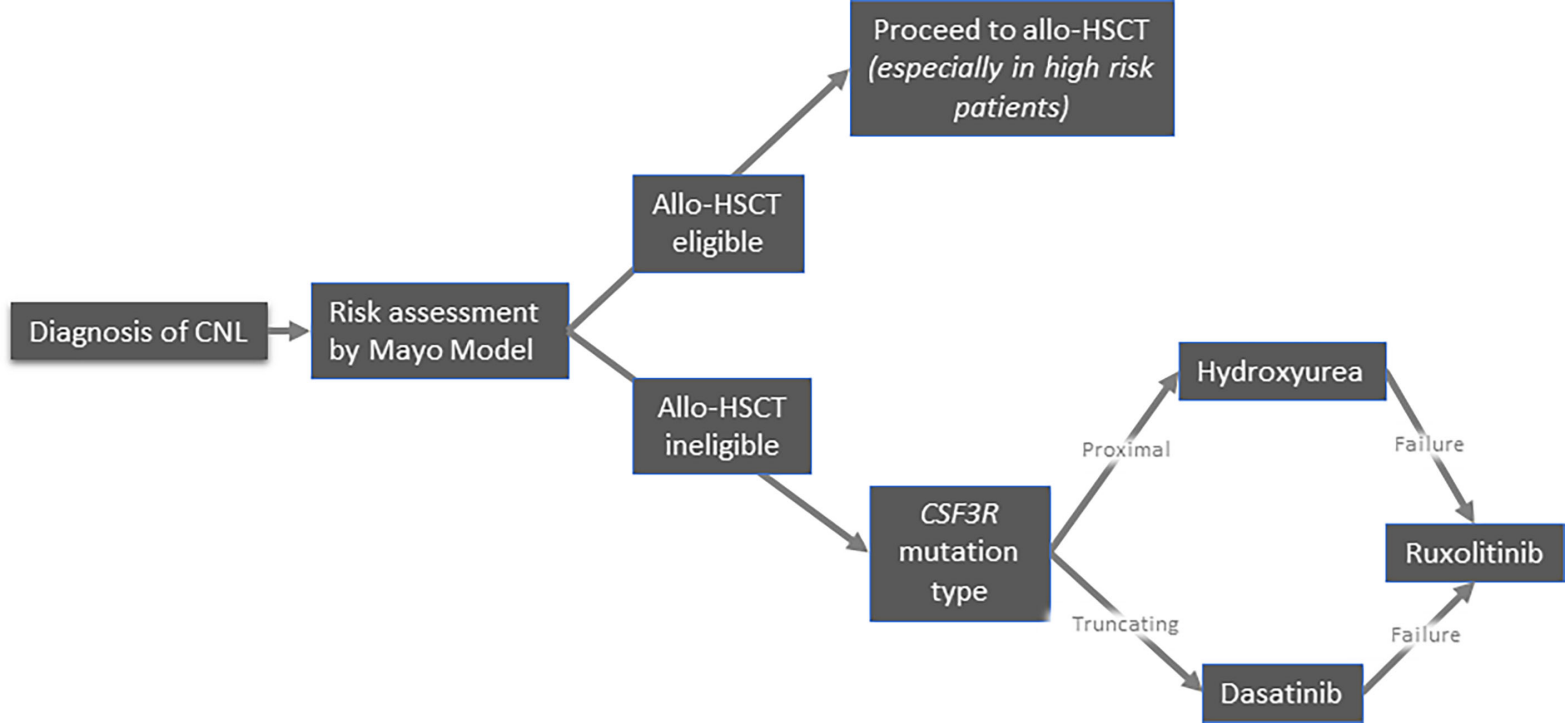
Suggested algorithm for treatment of patients with chronic neutrophilic leukemia.

Because of its exceptional rarity, there is no standard of care for CNL. Allogeneic hematopoietic stem cell transplantation (allo-HSCT) remains the only therapeutic approach with curative intent. Conventional treatments mainly aim to alleviate the disease burden, albeit they do not seem to affect the natural history of the disease and overall survival. Therefore, the development of potentially disease-modifying treatments constitutes a major unmet need. Currently, the available treatment options include pharmacological agents (hydroxyurea, interferons, JAK inhibitors, and other tyrosine kinase inhibitors) and allo-HSCT. A proposed algorithm of management of CNL is depicted in **Figure 4**.

### Hydroxyurea

Hydroxyurea, an oral cytoreductive agent, has traditionally been used in first-line treatment of CNL. Approximately 75% of patients are expected to demonstrate clinical response in terms of decrease in leukocytosis and/or splenomegaly; however, the responses are transient, as all patients are expected to demonstrate disease progression or transformation to AML within a median of 12 months (89).In this context, hydroxyurea should be considered palliative treatment, as it fails to halt clonal evolution and therefore disease progression.

### Interferon

Interferon-alpha (IFN-a) represents another therapeutic approach in CNL, based on published case reports; however, no clinical trial establishing its effectiveness has been reported. Although IFN-a might lead to long-term remission, even in patients primarily refractory to hydroxyurea, the impact of IFN-a to CNL clonal evolution has not been assessed. Recently, Yassin et al. reported the case of a woman with CNL achieving hematological response with pegylated interferon alpha-2a, albeit no data on duration of response have been disclosed (91). Despite the absence of solid evidence for the efficacy of IFN-a, it remains a valid option for CNL treatment.

### Intensive Chemotherapy

Conventional chemotherapy with anthracycline and cytarabine (3 + 7) might represent a treatment option for patients transforming to AML; however, overall survival is relatively poor, as expected for secondary AML (92). Regarding CNL in chronic phase, intensive chemotherapy is not recommended as it is accompanied by high treatment-related mortality and the majority of patients are refractory to this approach (11, 89).

### Ruxolitinib

The discovery of the key role of *CSF3R* in CNL and the JAK/STAT signaling pathway in CNL pathobiology has impelled the investigation of ruxolitinib, a JAK1/2 inhibitor, for the treatment of CNL. Preclinical studies demonstrated that ruxolitinib could decrease WBC count and splenomegaly in mice with *CSF3R*T618I mutated CNL; however, initial case reports demonstrated variable responses to ruxolitinib (93–96). Similarly, Szuber et al. in a case series of 19 patients, noted that ruxolitinib, received by four patients, was associated with a temporary response in half of the patients (41).

A phase II trial of ruxolitinib including 21 patients with CNL has been published recently. Overall response rate (ORR) was 58% for CNL patients. Among them, 4 complete remissions and 9 partial remissions were noted. Further, ORR was restricted to 8% in the group with wild-type *CSF3R*, while no association between response and cytogenetics, number of mutations, and *ASXL1* or *SETBP1* mutations was observed. Median OS for all patients was 18.8 months. By response, median survival for non-responders and responders was 15.6 and 23.1 months respectively. Most importantly, ruxolitinib was shown to decrease the *CSF3R* T618I allelic burden in responders, particularly for those achieving CR (mean absolute VAF change: −0.26 for CR, −0.05 for PR).

The effect of compound mutations of *CSF3R* on sensitivity to ruxolitinib has been an object of debate. *In vitro* studies have demonstrated the resistance of cell lines harboring compound mutation to ruxolitinib; however, Gunawan et al. have reported on a case with compound *CSF3R* mutations that had a substantial yet short-lived response to ruxolitinib (94). Most recently, Hinze et al. reported a case of a compound-mutated CNL achieving long-term remission with ruxolitinib (97). The co-occurrence of *CSF3R* and *SETBP1* mutations have shown to confer resistance to ruxolitinib; however, in the aforementioned phase II trial, no association between *SETBP1* mutation and response to ruxolitinib was noted (98).

The mechanisms underlying clonal evolutions in patients with CNL undergoing treatment with ruxolitinib might be multifaceted. Stoner et al. evaluated seven patients with CNL demonstrating molecular or clinical progression under treatment with ruxolitinib. VAF reduction of *CSF3R* mutation was noted in 3 cases, whereas two patients demonstrated no changes in allelic burden overtime. Primary resistance to ruxolitinib could be attributed to co-occurring *NOTCH2* and *SRSF2* mutations in the founder clone of one case. Notably, subclones harboring *STAT3*, *STAG2*, and *RUNX1* mutations emerged in three cases. Acting downstream of JAK kinases, *STAT3* could bypass the inhibitory effects of ruxolitinib, whereas co-operation of *RUNX1* with *CSF3R* mutations might be involved in CNL disease progression and AML transformation. On the contrary, the role of the *STAG2* mutations in disease progression has not been elucidated yet (99).

Fedratinib, another JAK inhibitor is being evaluated in a phase II trial for patients with CNL (NCT05177211).

### Other Tyrosine Kinase Inhibitors

The rationale for the use of dasatinib in CNL stems from the demonstration of the *in vitro* sensitivity of CNL lines harboring truncating *CSF3R* mutations to SRC kinase inhibition (20); however, no data on the *in vivo* efficacy of dasatinib are available. On the contrary, a recent case report demonstrated that co-occurrence of truncating and proximal *CSF3R* mutations confer resistance to dasatinib in a patient with CNL (97). In this context, a short trial with dasatinib could be offered in patients harboring truncating mutations with close monitoring for disease progression.

Preclinical data have suggested that compound mutations of *CSF3R* might exert their leukemogenic potential through enhancement of MAPK signaling. In support of this, trametinib, a MEK1/2 inhibitor, has demonstrated *in vitro* activity in compound-mutant CNL models; however, this agent has not been tested in clinical practice.

### Allogeneic Stem Cell Transplantation

Given the lack of therapeutic agents that could provide long-term disease control, allo-HSCT represents the only therapeutic option with curative potential; however, the published evidence remains scarce, consisting mostly of case reports and small case series. Elliot et al. reported of five patients with CNL who underwent allo-HSCT. Four of them achieved CR and remained disease-free up to six years from transplantation (89). In another case-series of 19 patients, two patients in blast transformation underwent allo-HSCT. One patient had a favorable outcome, remaining disease-free 40 months post-transplant, whereas the other patient died from transplantation-related complications (41).Ruan et al. in a population-based study reported that 2% of CNL patients underwent allo-HSCT. Notably, all of them were alive at five years post-treatment (90). Although these studies are informative of the curative role of allo-HSCT, they do not provide details pertaining to the transplantation procedures.

A retrospective nationwide study in Japan aimed to provide a more comprehensive overview of allo-HSCT in this rare myeloid neoplasm. Between 2003 and 2014, five patients with CNL underwent allo-HSCT. Three patients received hydroxyurea, one received dasatinib, and one was offered intensive chemotherapy, albeit none of the patients demonstrated response to treatment prior to transplantation. Notably, none of them received ruxolitinib. Four patients received transplantation from matched-unrelated donors, whereas one patient received transplantation from an HLA-haploidentical sibling donor. All but one patients received a myeloablative conditioning regimen. Neutrophil engraftment was achieved in all but one patient who died due to bleeding. Two of the patients who engrafted achieved CR that was retained without signs of relapse at day +362 and +441, whereas one patients, although in CR, died from sinusoidal obstruction syndrome at day +56; one patients showed no response to transplant and succumbed to the disease 76 days post-transplant.

## Conclusion

Chronic neutrophilic leukemia represents an extremely rare myeloproliferative neoplasm that has been poorly characterized for over a century, owing to the wide variability of the clinical presentation and laboratory findings of patients. Recently, there has been an increase in the understanding of the molecular pathogenesis of the disease, as mutations in the *CSF3R* gene are believed to be the driver mutations in most cases, albeit other mutations are required for the development of overt disease. The prognosis of the disease remains poor even for patients that proceed to allo-HSCT, which represents the only therapeutic option with curative intent. Studies on other therapeutic modalities such as ruxolitinib, have demonstrated satisfactory response rates; however, clonal evolution and emergence of resistance to these agents might limit their efficacy. Therefore, there is an unmet need for the optimal treatment of patients with CNL, highlighting the need for more studies enrolling patients with WHO-defined CNL, that could disentangle the complex genetic landscape of the disease and provide novel potential targets for development of more potent therapeutic agents.

## Author Contributions

Conceptualization, investigation, data curation, writing-original draft preparation: TPT, AS, AK, SGP, and VP. Writing—review and editing: TPT, and VP. Supervision: VP All authors have read, reviewed, edited and agreed to the published version of the manuscript.

## Conflict of Interest

The authors declare that the research was conducted in the absence of any commercial or financial relationships that could be construed as a potential conflict of interest.

## Publisher’s Note

All claims expressed in this article are solely those of the authors and do not necessarily represent those of their affiliated organizations, or those of the publisher, the editors and the reviewers. Any product that may be evaluated in this article, or claim that may be made by its manufacturer, is not guaranteed or endorsed by the publisher.
